# GASTROINTESTINAL MYELOID SARCOMA: A CASE REPORT

**DOI:** 10.51894/001c.122880

**Published:** 2024-08-30

**Authors:** Jacob Burch, Fady Banno, Justin Miller

**Affiliations:** 1 Gastroenterology Ascension Genesys

## 29

### INTRODUCTION

Myeloid sarcoma is a cancer that develops from myeloid cells and can manifest in any part of the body. Symptoms of myeloid sarcoma of the gastrointestinal (GI) tract may be vague or nonspecific making the diagnosis challenging.

### CASE DESCRIPTION

A 41-year-old female presented to the hospital complaining of one day of abdominal pain with associated nausea and vomiting. Computed tomography (CT) scan of her abdomen and pelvis with intravenous (IV) contrast was performed at an outside emergency department and was consistent with a small bowel obstruction. Conservative management with nil per os (NPO) and IV fluids was pursued. CT enterography was performed and revealed circumferential wall thickening consistent with acute enteritis involving a 10cm segment of the small bowel. This was believed to be caused by an infectious process. The patient’s symptoms improved after two days of conservative therapy, and she was discharged. At her follow-up appointment she complained of intermittent crampy abdominal pain. She was scheduled for an outpatient colonoscopy but was readmitted to the hospital with recurrent abdominal pain prior to this being performed. A repeat CT enterography showed progression of the circumferential wall thickening with slightly dilated loops of small bowel with air-fluid levels, indicating an obstruction. Diagnostic laparoscopy was performed, and a suspicious firm area of the bowel was found, leading to conversion to laparotomy. The mass was located within the mid-ileum, and a partial small bowel resection was performed. Initial pathology results were suggestive of intestinal T-cell lymphoma, but after a second opinion from an expert pathologist the final diagnosis was an extramedullary myeloid cell tumor (myeloid sarcoma).

### DISCUSSION

Myeloid sarcomas can occur in patients of any age and have a slight male predominance. Gastrointestinal tract involvement can present with abdominal pain, bloating, nausea, vomiting, changes in stool consistency, obstruction, or bleeding. Patients typically have a poor prognosis with a short life expectancy and high mortality rate. If primary myeloid sarcoma is not treated, it will almost always progress into acute myeloid leukemia (AML) within a few months. Treatment options include surgical excision, local radiation, and/or systemic chemotherapy depending on the tumor characteristics.

